# Macronutrient Intakes in 553 Dutch Elite and Sub-Elite Endurance, Team, and Strength Athletes: Does Intake Differ between Sport Disciplines?

**DOI:** 10.3390/nu9020119

**Published:** 2017-02-10

**Authors:** Floris Wardenaar, Naomi Brinkmans, Ingrid Ceelen, Bo Van Rooij, Marco Mensink, Renger Witkamp, Jeanne De Vries

**Affiliations:** 1 Sports and Exercise Studies, HAN University of Applied Sciences, 6525 AJ Nijmegen, The Netherlands; Naomi.brinkmans@han.nl (N.B.); Ingrid.ceelen@han.nl (I.C.); Bo.v.rooij@gmail.com (B.V.R.); 2 Division of Human Nutrition, Wageningen University, 6700 EV Wageningen, The Netherlands; Marco.mensink@wur.nl (M.M.); Renger.witkamp@wur.nl (R.W.); Jeanne.devries@wur.nl (J.D.V.)

**Keywords:** Dietary intake, sport nutrition, recommendations, guidelines, competitive athletes

## Abstract

Web-based 24-h dietary recalls and questionnaires were obtained from 553 Dutch well-trained athletes. The total energy and macronutrient intake was compared between discipline-categories (endurance, team, and strength) within gender, and dietary inadequacy, i.e., too low or high intakes, according to selected recommendations and guidelines, was evaluated by applying a probability approach. On average, 2.83 days per person were reported with a mean energy intake of 2566–2985 kcal and 1997–2457 kcal per day, for men and women, respectively. Between disciplines, small differences in the mean intake of energy and macronutrients were seen for both men and women. Overall, 80% of the athletes met the suggested lower-limit sport nutrition recommendation of 1.2 g·kg^−1^ of protein per day. The carbohydrate intake of 50%–80% of athletes was between 3 and 5 g·kg^−1^ bodyweight, irrespective of the category of their discipline. This can be considered as low to moderate, in view of their daily total exercise load (athletes reported on average ~100 min per day). In conclusion, only small differences in the mean energy and macronutrient intake between elite endurance, strength, and team sport athletes, were found. The majority of the athletes were able to meet the generally accepted protein recommendation for athletes, of 1.2 g·kg^−1^. However, for most athletes, the carbohydrate intake was lower than generally recommended in the existing consensus guidelines on sport nutrition. This suggests that athletes could either optimize their carbohydrate intake, or that average carbohydrate requirements merit a re-evaluation.

## 1. Introduction

It is commonly acknowledged that the average requirements for carbohydrate and protein in well-trained athletes, are above those set for the general sedentary population [1,2]. Indeed, recommendations for protein and carbohydrate intake for athletes, start slightly above estimated average requirement (EAR) values for the general population, i.e., 2.9 g·kg^−1^ bodyweight in the Netherlands [3]. However, current recommendations for carbohydrate and protein intake for athletes do show a considerable degree of variation [4], with carbohydrate intake recommendations ranging from 3 to 12 g·kg^−1^ per day, and protein recommendations from 1.2 to 2.0 g·kg^−1^ [5,6]. These wide ranges are set, especially for carbohydrate and protein recommendations, to allow athletes to fine-tune their personal needs to their total energy needs, specific training needs, and training performance [7]. In addition to these general values, tailored recommendations are set for specific sport disciplines, such as endurance, team, and strength sports [8,9,10]. Although athletes are considered a disciplined and motivated population, several recent studies have suggested that athletes may often not meet macronutrient recommendations [11,12,13,14,15,16,17]. However, this viewpoint has been questioned by others [18,19,20]. Differences in this viewpoint are partly due to different recommendations in the guidelines. Nowadays, a consensus has been reached on guidelines for carbohydrate intake; they should not be specified as a percentage of the contribution to total dietary energy intake (%TE), but in g·kg^−1^ [5]. This may have an impact on the conclusion as to whether or not athletes meet the recommendations.

Only a few studies have compared dietary intake, on multiple days, between elite athletes from different disciplines, such as endurance, team, and strength athletes [18,19]. Usually, endurance athletes have a higher energy intake, and it has been reported that they maintain substantially higher intakes of carbohydrate and protein than others. Thus, the idea that strength athletes would have a higher protein intake than other athletes, does not appear to have been confirmed [18,19,21]. Given the advancing insights into training methods, resulting in mixed training programs, i.e., including both endurance and strength training, especially in high level athletes, it leads researchers to question whether this differentiation in dietary intake between sport disciplines is still common practice.

The objective of the present study was to evaluate nutrition intake against generally accepted sport nutrition guidelines for carbohydrate and protein, and investigate whether differences exist in the macronutrient and energy intake between endurance, team, and strength athletes. This was assessed in a conditioning phase during a training period using a web-based 24-h recall method, with additional nutritional supplement questionnaires.

## 2. Materials and Methods

### 2.1. Study Design

The study was performed between February 2012 and June 2015. After enrolment, participants were asked to complete three or four unannounced web-based, 24-h dietary recalls, and an equal number of accompanying questionnaires about nutritional supplement use. All recalls were randomly scheduled during a two to four week period, on three non-consecutive days, not more than one week apart. At least two week days and one weekend day were included, and were a minimum of four days apart. If the collection (24-h recall and/or daily questionnaire) was not complete, an additional day was scheduled at the end of the research period. This gave athletes four opportunities to deliver at least two completed dietary assessments.

### 2.2. Study Population

Dutch elite and sub-elite athletes were recruited, based on their level of exercise and a minimum average exercise duration of at least 9 h a week. Participants were eligible for selection when they: (1) were granted an elite athlete status by the Dutch Olympic Committee (NOC*NSF) and/or; (2) had participated in a European or World Championship; and/or (3) had proven to be of a top level in their discipline or age group, on a national level. Elite athletes were first recruited by direct mailing through the Dutch Olympic Committee (NOC*NSF), and secondly, by sports dieticians working with this group of athletes. 

Written informed consent to participate was provided by all participants. For participants under the age of eighteen, informed consent was also obtained from their parents or guardian. No incentive was given for participation in the study. The survey was conducted according to the Declaration of Helsinki, as revised in 1983, and approved by the Medical Ethics Committee of Wageningen University.

### 2.3. 24-h Recalls and Questionnaires

All athletes were asked to fill in three web-based 24-h recalls using “Compl-eat™”, a program built by the Division of Human Nutrition of Wageningen University; except for a small group of male soccer players (*n* = 28), for whom four 24-h recalls and questionnaires were conducted face to face, by a trained dietician who precisely followed the web-based program. Compl-eat is based on the five-step multiple-pass method, a validated technique to increase the accuracy of dietary recalls [22]. This program also allows participants to select single foods and complete recipes, of which the amounts can be estimated by using household measures, standard portion sizes, weight in grams, or volume in liters [23]. Finally, the program probes the most common products that people tend to forget to mention. The program includes a wide selection of foods commonly used in a Dutch food pattern [24], but no dietary supplements or sport nutrition products, which were therefore the focus of a separate, web-based nutritional supplement questionnaire.

The web-based questionnaire asked participants to report their characteristics (i.e., gender, weight (kg), height (cm), and age in years), training load (total minutes of exercise per day), and nutritional supplement use, using the “Vitality portal”; a web-based questionnaire module built by the HAN University of Applied Sciences. Based on the question: “Did you use any dietary supplements, sport nutrition products, or ergogenic supplements yesterday?”, athletes were asked to provide details about all of the supplements which they had used during the previous day. They were able to select products and dosages out of a pre-specified list of more than 3400 dietary supplements, based on the Dutch database of dietary Supplements (NES) [25], and additional sports-specific dietary supplements and sport nutrition products. If a specific brand or product was missing, athletes were able to add this as a new product to the list. For each product that the athlete added, the specific brand, amount, and unit (dosage), was questioned. Afterwards, nutritional supplements were categorised by the researchers as dietary supplements (i.e., micronutrients and essential fatty acids), sport nutrition products (i.e., carbohydrate and protein based products), and ergogenic supplements (for example creatine, beta-alanine, and other products with ergogenic claims). Based on this input, the macronutrient composition and energy content was calculated, using the NES database.

Since response rates based on this questionnaire were considered too low, from 2013 onwards, they were completed via a telephone interview by a trained dietician, who followed the same steps as the original web-based questionnaire. Trained dieticians checked all web-based 24-h recalls and questionnaires for their completeness and unusual portion sizes, and processed all notes made by the participants. If information relating to food was missing, this was retrieved by contacting the relevant subjects. The adjustment of missing data, errors, and relevant notes were made in a standardised way, using standard portion sizes and recipes, according to a protocol [23]. We were able to retrieve the composition of almost all (>99%) of the added dietary supplements and sport nutrition products used, based on the label information provided by the manufacturers. If not, a product with a similar nutritional composition was selected.

### 2.4. Combining 24-h Recalls and Questionnaires

The reported food consumption was converted into energy and macronutrient intake, using the Dutch food composition database of 2010 (Stichting NEVO, 2010), the Dutch database for dietary Supplements (NES), and ingredient declarations of individual foods. After combining the results of both the 24 h recall and the questionnaire, the per person per day energy (MJ and kcal) and macronutrient intake (g.d^−1^, g·kg^−1^ and TE%: percentage contributions to total dietary energy intake) were calculated.

Data was preferably collected during a conditioning phase in the training program. Sports were categorized as endurance, team, and strength (-speed) sports, based on the characteristics of the sport. All team sports included were ball sports. Those sports with a high aerobic-endurance training character were characterized as endurance sports. Strength-speed sports were those sports that involved sprinting, or specialist sports, such as sailing and archery, which required static strength as part of the sports performance.

To reflect on the relationship between nutrient intake and energy expenditure, the total energy expenditure was estimated (eTEE) for each group of athletes, per sport and discipline. This was done by applying the formula of Schofield [3], which permits an estimation of the BMR, excluding the hours of exercise per day. This value was multiplied by a physical activity level (PAL) of 1.4 [26], and finally, the energy expenditure based on minutes of exercise, using the tables of Ainsworth, was added to the value [27].

### 2.5. Statistical Analyses

Statistical analysis (SPPS version 23) was carried out for participants with at least two-day dietary assessments. Results were checked for normal distribution, using histograms for curves, skewness, and kurtosis. No log transformation was needed. The intake distribution was adjusted for day to day variation, using the formula SD_corrected_ = SD_observed_ * √r_ic_, where r_ic_ is the intra class correlation coefficient (ᵖ = ᵟ_s_^2^/ ᵟ_s_^2^ + ᵟ_e_^2^). The mean ± SD_corrected_ of dietary intake was reported for the different subgroups. Within and between the subject coefficients of variation (CV_W_ and CV_b_) for energy and macronutrients, the results were similar to those which have previously been found [28,29]. The precision of the mean estimate (D_t_) was below 12.5% for all macronutrients [30].

One-way ANOVA was performed to determine the differences within gender, for energy, macronutrients, dietary fibre, and fluid intake. Statistical significance was set at *p* ≤ 0.05, and the main effect was subsequently analyzed using a Bonferroni corrected post-hoc test. 

The proportion (%) of athletes at risk of inadequate intake, was estimated using the probability approach. The probability approach accounts for the probability of inadequacy P(r_i_) for each individual, and the distribution of the nutrient intake (exposure), i.e., the probability of exposure P(x_i_) for each level of exposure. The P(r_i_) is obtained by filling in the *z*-scores (*z*_i_) for the usual intake, minus the EAR or sport nutrition recommendation, and divided by the SD of the requirement, e.g., (x_i_ − EAR)/Sd_req_). The P(x_i_) is provided by the frequency distribution of nutrient intake in the population, assuming a normal distribution. The prevalence of inadequacy is than calculated as P_inadequacy_ = ∑P(x) * P(r_i_). The estimated average requirements (EAR) used were 0.6 g·kg^−1^ for protein and 2.9 g·kg^−1^ for carbohydrate [3]. Furthermore, the generally accepted sport nutrition recommendations for protein, of 1.2 g·kg^−1^ [31], and for carbohydrates, of 5.0 g·kg^−1^, were considered [5]. 

The ratio of calculated energy intake/BMR using Schofield’s formula [32], may be referred to as the food intake level (FIL) value. Compared with the physical activity level (PAL), the ratio FIL/PAL should equal 1.0, if there is no bias in the estimation of the energy intake. We used a lower limit Physical Activity Level (PAL) of 1.55, as was previously employed in our validation study [33].

## 3. Results

A total of 759 Dutch elite and sub-elite competitive athletes fulfilled the inclusion criteria, and were included in the study. Due to incomplete 24-h dietary recalls or dietary questionnaires, 206 athletes were excluded, and only the data of the remaining 553 athletes were analyzed (Table 1). Athletes held an elite athlete status of the Dutch Olympic Committee (NOC*NSF) (*n* = 195); or had participated in a European League (soccer) or European/World Championship (*n* = 60); or had proven to be of a top level in their discipline or age group on a (inter)national level (*n* = 298). The study population consisted of both male (59%) and female (41%) athletes, representing 20 different sports. They were mostly young-adult elite athletes (mean ± SD: 20.5 ± 4.5 years for men and 20.7 ± 5.4 years for women). Athletes were categorized into three disciplines, i.e., endurance, team, and strength sports. On average, strength athletes reported the highest number of training hours, followed by endurance athletes, and then team athletes, in both men and women (see Table 1). Participants reported an average of 2.83 days per person; 2.89 days for men and 2.75 days for women.

### 3.1. Energy Intake

For men, as shown in Table 2, the mean estimated energy intake, including nutritional supplements, ranged between 2561 and 2994 kcal per day. For women, the mean energy intake ranged between 1997 and 2457 kcal per day (Table 3). The inclusion of nutritional supplements to the basic dietary intake added on average ~70 kcal to the energy intake of both men and women. This mean difference, between including or excluding nutritional supplements, was small but significant for both men and women, within all sports categories (*p* < 0.01).

For men, the median food intake level (FIL) values, as an indicator of misreporting, was 1.60 for endurance athletes, which was above the estimated minimum cut-off limit for PAL, of 1.55. However, both team and strength male athletes reported below this level (FIL of 1.4). For women, the median FIL values of the endurance athletes (1.57) was also above a PAL of 1.55, but both team (1.31) and strength athletes (1.39) scored below this cut-off limit.

### 3.2. Differences between Endurance, Team, and Strength Athletes

Male endurance athletes reported a significantly higher level of intake of energy and all macronutrients, when compared to male team sports athletes (Table 2). Male strength athletes consumed higher amounts of protein and dietary fibre, than athletes active in team sports. No differences were seen between endurance and strength athletes.

Similar to men, female endurance athletes reported a significantly higher intake of energy and macronutrients, compared to team sports athletes, but female endurance athletes also consumed more energy, carbohydrates, and fibre, than strength athletes (Table 3). In females, no differences were seen between team and strength athletes.

With regard to the contribution of macronutrients to the total energy intake (TE%), no differences were seen in carbohydrate and fat values between sport disciplines, in both men and women (Table 2 and Table 3). In females, the contribution of protein to the total energy was higher in both strength (18.4 TE%) and team sports athletes (17.8 TE%), when compared to endurance athletes (16.4 TE%), while in men, only strength athletes and endurance athletes significantly differed: 18.0 and 16.1 TE% protein intake, respectively (Table 2).

Differences, for both men and women, were also seen when carbohydrate and protein intake were expressed as g·kg^−1^ (Table 4). In men, endurance and team athletes consumed more carbohydrates than strength athletes, while in women, the endurance athletes consumed more carbohydrates than both strength and team athletes, but there were no differences in carbohydrate intake between strength and team athletes.

In women, team athletes consumed less protein per kg bodyweight than endurance and strength athletes (Table 5); in men, no differences were seen.

### 3.3. Carbohydrate and Protein Intake as Compared to Reference Values

When sport nutrition products were included in the analysis, not all of the athletes met the EAR for carbohydrates (2.9 g·kg^−1^). For men, the prevalence of an intake below the EAR value was 5.5%, 8.0%, and 13.6%, for endurance, team, and strength athletes, respectively. For women, a higher prevalence of low intake was seen, of 6.1%, 26.0%, and 17.4%, for endurance, team, and strength athletes, respectively. When carbohydrate intake was compared with a moderate sports nutrition recommendation of 5 g·kg^−1^ carbohydrate per day (Table 4), the prevalence of inadequacy for carbohydrates among men was 48.3% for endurance athletes, compared to 50.5% for team athletes and 96.2% for strength athletes. In women, the prevalence of inadequate intake of carbohydrates was 54.4% for endurance sports, 80.5% for team sports, and 73.0% for strength sports, when compared to the sports nutrition recommendation of 5 g·kg^−1^ per day. Including sport nutrition products in the analysis resulted in a somewhat lower prevalence of intakes below the recommendations.

For protein, our results confirmed that all athletes (>98%) met the estimated average requirement (EAR) for protein (0.6 g·kg^−1^), when sport nutrition products were included. When protein intake was compared with the sports nutrition recommendation of about 20%–30%, the prevalence of intakes below 1.2 g·kg^−1^ protein, were seen in all disciplines, for both men and women (Table 5). The only exception was the female team athletes group, showing a prevalence of inadequate intake of 41.8%, even when nutritional supplements were included.

## 4. Discussion

This study provides an insight into the nutritional intake of a unique, large cohort of elite and sub-elite athletes (*n* = 553, 327 men and 226 women), practicing a wide variation of sports. Small differences were found in the absolute mean energy and macronutrient intake between athletes practising different sporting disciplines. In general, the mean intake of energy, carbohydrate, and protein was higher in endurance athletes, when compared to team athletes and, to some extent, to strength athletes. When expressed in g·kg^−1^, both male endurance and team athletes reported higher carbohydrate intakes than strength athletes. Female endurance athletes not only reported a higher carbohydrate intake than strength athletes, but also than team athletes. For the protein intake, no differences were seen for men between the different athlete groups, but female endurance and strength athletes both reported higher protein intakes than team athletes, when calculated as g·kg^−1^. Almost all of the athletes met the EAR for protein, but a substantial number of athletes (20%–30%) reported carbohydrate intakes below the EAR. Except for female team athletes, all other disciplines reported a protein intake of ~20% below the generally accepted sport nutrition recommendation of 1.2 g protein per kilogram bodyweight per day. Additionally, more than half of the athletes did not reach the generally accepted sport nutrition recommendation for carbohydrate intake of 5.0 g per kilogram bodyweight per day.

Regarding the carbohydrate intake of athletes, recent literature data are scarce, and they give a diverse picture. Moreover, the number of large surveys comparing dietary intake and adequacy in different sports disciplines, is limited [18,19,34,35,36]. Of these, only two recent studies categorized athletes in a manner comparable to our study [18,19]. In line with our data, both studies reported higher carbohydrate intake levels for endurance athletes, when compared to other types of athletes. However, absolute levels, as reported by Burke et al. [18], for endurance athletes were higher (6.8 g·kg^−1^, mix of men and women) in comparison to the 5.2 and 5.0 g·kg^−1^ for men and women that we observed. While the reported carbohydrate intakes of endurance athletes in the present study were somewhat lower compared to others [28], the carbohydrate intakes of team athletes [10] and strength athletes [8] were in line with previously reported intakes. Although it was suggested that women appear to be less reliant on glycogen during exercise and glycogen recovery [37], the carbohydrate intake in g·kg^−1^ appeared to be similar for both men and women performing team sports. This would suggest that women performing the same type of sport were consuming relatively more carbohydrates than their male counterparts. 

Our finding, that carbohydrate intake levels were lower than expected, and lower than previously reported in the literature [18,19], deserves some attention. In our view, the large number of participants practicing different sports, and the fact that we collected data for on average of 2.83 reporting days, resulted in a representative picture of this population. Also, information on daily variation in intake can be extracted from our data. Our results suggest that peaks in carbohydrate intake on individual days were limited in most of our athletes. We expected athletes to differentiate in carbohydrate intake, resulting in low, moderate, and high intakes as part of the data collected, but high single daily carbohydrate intakes above 8 g·kg^−1^ per athlete were exceptional (<6% of all days collected). However, despite the fact that some degree of underreporting may have affected our data, Timon et al. (2016) stated that web-based 24-h recall tools generally corroborated the respective reference values for intakes of macronutrients. The combined method of web-based 24-h recalls and questionnaires was previously validated by our research group in a sub-sample of this population [33]. Based on that study, it was concluded that the method was suitable for the ranking of athletes based on their intake (r = 0.65 (95%CI: 0.45–0.79)), with an acceptable underreporting rate of 25.5% [33]. Based on the estimated TEE, it seems likely that underreporting was comparable in the current study. To further investigate this possibility of under-reporting in the current population, we compared the food intake level (FIL) to the estimated lower limit PAL for this population, of around 1.55; equal to our previously published validation study [33]. The median FIL found in the present study was 1.53 for men and 1.43 for women, which indicates possible energy under-reporting, as in line with our previously reported validation study (FIL of 1.52) [33]. These median FIL values were improved by the higher FIL values of endurance athletes, as other categories for both men and women reported much lower values. The relatively high under-reporting by team and strength athletes, in comparison to endurance athletes, may have affected our results. If we assume that nutrient intake was not biased by selective under-reporting, and that their energy requirements were more or less comparable, we can estimate that the degree of under-reporting for carbohydrates and protein was equal to energy under-reporting. If this is taken into account, our data indicate that no differences in the mean values are present between sport disciplines, for both men and women. This added to our observation that differences in estimated TEE between disciplines, were found to be small, suggesting that macronutrient intakes within each gender are very similar for the different disciplines. This also confirms the idea that, due to advancing insights in training methods, resulting in mixed training programs, i.e., including both endurance and strength training, especially in high level athletes, differences in dietary intake between sporting disciplines are, in practice, not present.

Although the contribution of carbohydrates to the total energy intake was >50 energy % in all sporting disciplines, the absolute carbohydrate intake was still not very high in our population, even if possible under-reporting is taken into account. These findings raise the important question of whether carbohydrate intake levels are actually sub-optimal in the light of current recommendations. In our population, a basic diet was the main source of carbohydrates, as intake through sport nutrition products was limited. From a physiological perspective, it seems advantageous to consume over 5–10 grams of carbohydrates per kg of body weight, for most of these athletes. Athletes who exercise >90 min a day or perform intermittent exercise, particularly benefit from sufficient carbohydrate intake. These dietary carbohydrates are mainly used to restore muscle and liver glycogen, enhancing recovery between (multiple) training sessions or competitive events [5]. From this, it could be concluded that their carbohydrate intake needs to be improved [38]. However, whether a low carbohydrate intake, both basal and during exercise, negatively affects direct and long term athletic performance, is currently under debate [39,40].

The question is thus raised of whether athletes, for example, those who are involved in prolonged sub maximal exercise, should always consume a high carbohydrate diet [39]. Moreover, it has been proposed that athletes can adapt to lower muscle glycogen stores [41]. This can be achieved with moderate carbohydrate intake (3–5 g·kg^−1^ per day, such that it does not necessarily impair training or competition outcomes) [41]. If this is the case, athletes with a moderate carbohydrate intake in the present study, are not necessarily at risk of impaired performance capacity. On the other hand, as daily carbohydrate intake is not static, a low to moderate average for carbohydrate intake is still leaving an opportunity for athletes to consume high amounts of carbohydrate, tailored according to the fuel cost of the training load and carbohydrate requirements, when needed [5]. 

The reported protein intake in our athletes was comparable to previously reported data from Burke et al. [18], and slightly lower than those reported by De Sousa et al. [19]. It remains difficult to compare the quality of protein intake relative to recommendations, with previous reports, due to the fact that different recommendations, between 1.2 and 1.4 or 1.5 g·kg^−1^, were used [11,13]. Although some differences were seen for protein intake between sporting disciplines in our study, most athletes (~70%) had a daily protein intake of above 1.2 g·kg^−1^. As recommendations were mostly met, the observed differences between groups of athletes may be of less relevance. It is worth mentioning that the average protein intake of the general Dutch population is already relatively high (~1.0 and ~1.3 g·kg^−1^ for females and males in the age of 19–30 years respectively), which probably underlies our observation [42]. Although the absolute protein intake seems sufficient in most athletes, a substantial sample of the athletes did not meet this recommendation. Furthermore, it is possible that protein timing over the day needs further attention in all athletes, to optimize adaptation and muscle protein synthesis [43].

This study has some limitations. First, athletes were asked to self-report body weight. This may have introduced some error into the calculated results of carbohydrate and protein intake in g·kg^−1^. Furthermore, for 1% of all 932 dietary supplements and sport nutrition products collected, we were not able to retrieve the original ingredient declaration. Therefore, we used a reference product, which could have affected, although to a small extent, the estimated macronutrient distribution. In addition, the inclusion of athletes and the way in which their disciplines were classified into endurance, team, and strength sports, leaves some room for discussion. For example, we chose to categorize swimming as an endurance sport, despite the fact that most swimmers were competing in sprint disciplines during competitions; we did this because of the high aerobic workload of the training program. Besides this, the strength athletes included in the study do not represent the full scope of strength sports. The participating strength athletes mostly performed sports that required speed, in combination with strength or static strength. Therefore, the generalizability of the results, especially in relation to strength athletes, should be handled with care.

## 5. Conclusions

Only small differences exist in the mean energy and macronutrient intake between elite endurance, strength, and team sports athletes. This confirmed the idea that, due to advancing insights into training methods, resulting in mixed training programs, differences in dietary intake between sport disciplines are, in practice, not present. Furthermore, the majority of the athletes were able to meet the generally accepted protein recommendation for athletes, of 1.2 g·kg^−1^, but carbohydrate intake was low to moderate in most athletes, with a value between 3–5 g·kg^−1^. Considering the existing consensus on sport nutrition carbohydrate recommendations, i.e., ~5 g·kg^−1^ day, our data suggest that a substantial part of athletes across all disciplines should either improve their carbohydrate intake to meet recommendations, or that a re-evaluation of average carbohydrate needs and related recommendations is merited.

## Figures and Tables

**Table 1:** Mean group characteristics ± SD for type of sport, and exercise divided by gender.

	Men							Women						
	*n*	Height (cm)	Weight (kg)	BMI (kg^−1^·m^2^)	Age (Year)	Exercise (min)	eTEE (MJ)	*n*	Height (cm)	Weight (kg)	BMI (kg^−1^·m^2^)	Age (Year)	Exercise (min)	eTEE (MJ)
**Endurance**	**157**	**184.2 ± 7.5**	**75.1 ± 10.8**	**22.0 ± 2.1**	**29.1 ± 13.7**	**92.5 ± 52.5**	**14.0**	**83**	**173.3 ± 7.6**	**63.3 ± 8.4**	**21.0 ± 2.1**	**23.9 ± 10.1**	**104.3 ± 59.0**	**12.67**
Rowing ●▲△	34	191.5 ± 6.5	85.1 ± 10.3	23.1 ± 1.8	21.6 ± 2.2	88.9 ± 34.7	14.9	26	178.7 ± 7.1	70.8 ± 5.9	22.2 ± 1.4	21.8 ± 2.4	107.2 ± 55.7	13.3
Swimming ●○	11	188.0 ± 7.0	82.1 ± 8.4	23.2 ± 1.8	20.1 ± 4.8	88.0 ± 35.6	14.9	9	174.6 ± 7.5	63.8 ± 5.5	20.9 ± 1.0	16.9 ± 1.7	105.4 ± 37.1	12.9
Ice skating ○▲	15	181.8 ± 5.6	72.0 ± 6.6	21.7 ± 1.2	18.8 ± 1.9	97.4 ± 85.1	13.4	11	171.4 ± 4.4	60.8 ± 5.8	20.7 ± 1.5	17.3 ± 2.4	97.5 ± 68.4	11.8
Road cycling ○▲	34	180.4 ± 7.1	66.1 ± 7.3	20.3 ± 1.7	18.3 ± 4.4	105.9 ± 57.0	12,9	14	172.3 ± 6.3	63.4 ± 5.9	21.4 ± 1.9	24.2 ± 7.2	128.0 ± 71.8	12.6
Running(mila) ○▲	8	182.1 ± 4.8	66.2 ± 3.7	20.0 ± 1.1	19.1 ± 3.1	85.5 ± 30.7	14.6	11	171.3 ± 6.5	52.6 ± 6.1	17.9 ± 1.7	18.3 ± 4.0	93.5 ± 51.7	12.6
Ultraendurance ●△	55	182.3 ± 6.0	75.1 ± 8.7	22.6 ± 1.9	46.4 ± 6.6	86.8 ± 53.1	13.9	12	165.3 ± 4.8	58.8 ± 6.4	21.5 ± 2.4	44.3 ± 9.3	85.5 ± 61.7	11.2
**Team**	**138**	**175.8 ± 12.4**	**67.0 ± 15.3**	**21.3 ± 2.7**	**17.6 ± 5.0**	**64.4 ± 37.1**	**11.9**	**104**	**173.6 ± 7.8**	**67.0 ± 7.5**	**22.2 ± 2.0**	**20.8 ± 4.8**	**98.3 ± 57.4**	**13.0**
Soccer youth ▲	63	168.2 ± 12.8	55.5 ± 13.2	19.3 ± 2.1	14.0 ± 1.7	52.8 ± 29.0	10.3	16	168.4 ± 6.6	58.4 ± 6.1	20.6 ± 1.5	15.8 ± 0.8	61.4 ± 24.6	10.7
Soccer talent ▲	26	179.8 ± 6.2	71.1 ± 5.8	22.0 ± 1.5	17.8 ± 1.6	56.1 ± 32.0	12.2	-	-	-	-	-	-	
Soccer prof	30	182.0 ± 7.3	78.3 ± 8.7	23.6 ± 1.6	22.9 ± 4.1	78.9 ± 28.1	13.5	-	-	-	-	-	-	
Volleyball ●○	-	-	-	-	-	-		18	184.6 ± 5.8	72.6 ± 5.9	21.3 ± 1.2	18.1 ± 3.7	134.1 ± 70.8	12.6
Water polo ▲	12	189.1 ± 7.5	85.6 ± 8.5	23.9 ± 2.0	23.1 ± 8.0	116.5 ± 57.6	17.9	12	174.6 ± 3.0	70.3 ± 6.6	23.1 ± 2.3	21.4 ± 4.3	136.7 ± 72.1	16.0
Rugby Sevens ●○	-	-	-	-	-	-		29	169.0 ± 5.4	66.5 ± 6.1	23.3 ± 1.9	24.0 ± 5.0	85.2 ± 50.7	13.1
Hockey ○	7	180.9 ± 7.5	73.7 ± 7.0	22.5 ± 0.9	18.5 ± 1.0	47.1 ± 5.7	12.4	11	169.3 ± 3.8	61.7 ± 4.7	21.5 ± 1.5	18.5 ± 0.8	76.7 ± 25.6	11.7
Handball ●	-	-	-	-	-	-		18	176.6 ± 4.4	70.7 ± 5.3	22.7 ± 1.5	24.1 ± 3.1	103.9 ± 46.2	13.6
**Strength**	**32**	**182.7 ± 5.5**	**82.3 ± 9.2**	**24.6 ± 2.6**	**21.2 ± 4.9**	**110.5 ± 85.4**	**15.0**	**39**	**166.4 ± 9.2**	**61.1 ± 10.5**	**21.9 ± 2.2**	**20.9 ± 6.7**	**150.9 ± 94.5**	**11.7**
Track cycling ●	5	186.2 ± 6.3	88.5 ± 6.6	25.5 ± 1.4	19.6 ± 1.1	191.7 ± 53.5	19.9	6	172.4 ± 6.8	70.6 ± 10.6	23.6 ± 2.1	19.3 ± 1.2	169.4 ± 54.4	13.9
BMX ●○	12	180.6 ± 5.3	77.8 ± 10.0	23.8 ± 2.3	19.2 ± 3.3	100.3 ± 88.1	14.7		“	“	“	“	“	
Sprint/bobsled ●○▲	4	184.5 ± 3.0	84.6 ± 8.9	24.9 ± 3.0	21.5 ± 1.3	74.6 ± 27.3	14.2	8	171.6 ± 4.9	63.5 ± 5.7	21.6 ± 1.5	20.8 ± 3.1	62.7 ± 29.1	11.2
Cross fit △	5	184.7 ± 4.0	88.6 ± 5.4	26.0 ± 2.1	25.6 ± 7.8	41.3 ± 24.0	13.3	6	167.2 ± 5.8	63.3 ± 5.4	22.6 ± 1.3	31.5 ± 9.6	90.0 ± 45.7	11.3
Sailing ●	-	-	-	-	-	-	-	6	171.6 ± 7.5	64.8 ± 8.3	22.0 ± 2.3	23.0 ± 2.2	163.7 ± 81.9	10.9
Gymnastics ●○	-	-	-	-	-	-	-	13	157.7 ± 8.0	52.4 ± 10.2	20.8 ± 2.6	16.0 ± 3.0	218.7 ± 103.0	11.2
Archery ●	6	181.3 ± 6.8	79.2 ± 7.3	24.3 ± 3.9	22.4 ± 6.3	145.1 ± 104.2	13.0	-	-	-	-	-	-	

Symbols indicate the level of athletes included: ● = national team competing at international level, ○ = national youth team competing at international level, ▲ = team competing at national level, △ recreational level competing mainly at national level. Women Track cycling and BMX are combined. eTEE = Estimated Total Energy Expenditure, no SD was calculated because this was only an indicative estimation per sport. “ = combining to sports (track cycling and BMX women), - = no participants in this category.

**Table 2:** Total mean intake, including nutritional supplements, of energy, macronutrients, fluid, and dietary fibre in men (*n* = 327).

	Absolute Mean Intake							Macronutrient Contribution for Total Energy (TE%)		
	Energy (Kcal)	Energy (MJ)	CHO (g)	PRO (g)	FAT (g)	Fluid (mL)	Fibre (g)	CHO *	PRO	FAT
**Endurance (*n* = 157)**	**2994 ± 556** ★	**12.6 ± 2.3** ★	**390 ± 76** ★	**117 ± 28** ★	**96 ± 20** ★	**2915 ± 788** ★	**34 ± 7** ★	**55.1**	**16.1** ◆	**28.8**
Rowing (*n* = 34)	3784 ± 593	15.9 ± 2.5	489 ± 76	156 ± 31	119 ± 19	3628 ± 839	45 ± 6	54.8	17.4	27.8
Swimming (*n* = 11)	3076 ± 638	12.9 ± 2.7	413 ± 84	130 ± 16	92 ± 26	2624 ± 518	36 ± 7	56.2	17.2	26.6
Ice skating (*n* = 15)	2904 ± 481	12.2 ± 2.0	381 ± 77	113 ± 21	98 ± 14	2480 ± 533	31 ± 3	54.2	15.8	30.0
Road cycling (*n* = 34)	2735 ± 444	11.5 ± 1.9	363 ± 55	111 ± 18	85 ± 18	2389 ± 733	31 ± 6	56.4	16.4	27.1
Running (mila) (*n* = 8)	2946 ± 317	12.4 ± 1.3	419 ± 87	110 ± 11	82 ± 10	3160 ± 735	39 ± 9	59.3	15.4	25.3
Ultra endurance (*n* = 55)	2681 ± 239	11.3 ± 1.0	337 ± 35	95 ± 11	90 ± 18	2941 ± 643	29 ± 3	54.6	15.0	30.4
**Team (*n* = 138)**	**2561 ± 395** ★	**10.8 ± 1.7** ★	**327 ± 56** ★	**104 ± 21** ★■	**85 ± 14** ★	**2455 ± 791** ★	**24 ± 4** ★■	**53.8**	**16.5**	**29.7**
Soccer youth (*n* = 63)	2275 ± 211	9.6 ± 0.9	303 ± 32	82 ± 8	76 ± 13	1805 ± 232	23 ± 2	56.0	14.7	29.3
Soccer talent (*n* = 26)	2700 ± 300	11.4 ± 1.3	361 ± 66	114 ± 13	83 ± 3	2692 ± 464	252 ± 2	55.1	17.3	27.6
Soccer prof (*n* = 30)	2841 ± 398	11.9 ± 1.7	341 ± 51	135 ± 13	95 ± 14	3550 ± 743	262 ± 5	50.2	19.4	30.4
Water polo (*n* = 12)	3055 ± 542	12.8 ± 2.3	373 ± 86	120 ± 20	104 ± 20	2825 ± 715	265 ± 8	52.5	16.9	30.7
Hockey (*n* = 7)	2566 ± 138	10.8 ± 0.6	274 ± 25	109 ± 10	105 ± 14	2102 ± 361	218 ± 1	46.0	17.0	37.0
**Strength (*n* = 32)**	**2846 ± 395**	**12.0 ± 1.7**	**348 ± 61**	**127 ± 24** ■	**94 ± 28**	**2815 ± 475**	**32 ± 6** ■	**52.3**	**18.0** ◆	**29.8**
Track cycling (*n* = 5)	3212 ± 225	13.5 ± 0.9	400 ± 21	150 ± 29	99 ± 9	2759 ± 398	32 ± 1	52.2	19.0	28.8
BMX (*n* = 12)	2739 ± 696	11.5 ± 2.9	383 ± 85	109 ± 27	76 ± 38	2599 ± 783	35 ± 10	59.4	16.0	24.6
Sprint/bobsled (*n* = 4)	2969 ± 127	12.5 ± 0.5	369 ± 15	132 ± 3	99 ± 12	3352 ± 110	35 ± 2	52.5	17.6	29.9
Cross fit (*n* = 5)	3308 ± 172	13.9 ± 0.7	300 ± 53	161 ± 12	144 ± 17	3314 ± 290	29 ± 5	40.6	19.8	39.6
Archery (*n* = 6)	2291 ± 288	9.6 ± 1.2	263 ± 59	111 ± 17	81 ± 3	2519 ± 556	25 ± 7	47.7	19.7	32.6

Intake values are expressed as mean intake ± SD; Differences between categories are indicated by symbols (one-way ANOVA: *p* < 0.05, with post-hoc Bonferroni correction), each comparison between groups has its own symbol; ★ Endurance vs. Team, ◆ Endurance vs. Strength and ■ Team vs. Strength; No analysis was performed for testing differences between sports within the categories for endurance, team, and strength sports; * TE% for CHO includes estimated TE% of dietary fibre.

**Table 3:** Total mean intake, including nutritional supplements, of energy, macronutrients, fluid and dietary fibre in women (*n* = 226).

	Absolute Mean Intake							Macronutrient Contribution for Total Energy (TE%)		
	Energy (Kcal)	Energy (MJ)	CHO (g)	PRO (g)	FAT (g)	Fluid (mL)	Fibre (g)	CHO *	PRO	FAT
**Endurance (*n* = 83)**	**2459 ± 520** ★◆	**10.3 ± 2.2** ★◆	**312 ± 58** ★◆	**97 ± 19** ★	**81 ± 28** ★	**2842 ± 930** ★	**32 ± 8** ★◆	**54.2**	**16.4** ★◆	**29.5**
Rowing (*n* = 26)	2909 ± 662	12.2 ± 2.8	362 ± 50	112 ± 23	99 ± 45	3228 ± 657	39 ± 10	54.2	16.1	29.7
Swimming (*n* = 9)	2495 ± 335	10.5 ± 1.4	341 ± 41	105 ± 13	70 ± 7	2429 ± 652	32 ± 3	56.8	17.2	26.0
Ice skating (*n* = 11)	2224 ± 424	9.3 ± 1.8	283 ± 61	85 ± 11	78 ± 12	2209 ± 468	26 ± 3	53.2	15.8	31.0
Road cycling (*n* = 14)	2127 ± 363	8.9 ± 1.5	264 ± 53	92 ± 14	69 ± 13	3121 ± 1324	30 ± 9	53.1	17.9	29.0
Running(mila) (*n* = 11)	2299 ± 102	9.7 ± 0.4	297 ± 25	96 ± 5	73 ± 10	2429 ± 977	30 ± 6	53.7	17.3	29.0
Ultra endurance (*n* = 12)	2205 ± 288	9.3 ± 1.2	280 ± 61	76 ± 15	75 ± 12	2949 ± 1080	29 ± 8	54.6	14.3	31.1
**Team (*n* = 104)**	**1997 ± 201** ★	**8.4 ± 0.9** ★	**247 ± 32** ★	**87 ± 10** ★	**66 ± 11** ★	**2441 ± 631** ★	**24 ± 5** ★	**52.2**	**17.8** ★	**30.0**
Soccer youth (*n* = 16)	1965 ± 293	8.3 ± 1.2	256 ± 31	76 ± 4	66 ± 13	1695 ± 255	20 ± 5	54.2	15.8	30.0
Volleyball (*n* = 18)	1998 ± 86	8.4 ± 0.4	260 ± 12	87 ± 5	59 ± 9	2585 ± 485	27 ± 3	55.6	17.8	26.6
Water polo (*n* = 12)	1873 ± 111	7.9 ± 0.5	245 ± 29	75 ± 7	58 ± 12	2087 ± 389	24 ± 7	55.6	16.4	28.0
Rugby Sevens (*n* = 29)	2056 ± 207	8.6 ± 0.9	244 ± 44	93 ± 0	71 ± 10	2794 ± 594	23 ± 3	49.9	18.5	31.6
Hockey (*n* = 11)	2091 ± 373	8.8 ± 1.6	259 ± 37	89 ± 10	70 ± 20	2216 ± 562	26 ± 3	52.9	17.5	29.6
Handball (*n* = 18)	1955 ± 224	8.2 ± 1.0	222 ± 21	92 ± 27	69 ± 3	2767 ± 617	24 ± 8	48.0	19.4	32.6
**Strength (*n* = 39)**	**2073 ± 417** ◆	**8.7 ± 1.7** ◆	**251 ± 46** ◆	**92 ± 23**	**69 ± 21**	**2447 ± 866**	**25 ± 6** ◆	**51.8**	**18.4** ◆	**29.8**
Track cycling/BMX (*n* = 6)	2202 ± 46	9.3 ± 0.2	285 ± 13	97 ± 18	67 ± 2	2385 ± 885	26 ± 4	54.0	17.9	28.1
Sprint/bobsled (*n* = 8)	2269 ± 401	9.5 ± 1.7	297 ± 58	88 ± 16	75 ± 19	2514 ± 778	24 ± 6	54.4	16.4	29.2
Cross fit (*n* = 6)	2609 ± 329	10.9 ± 1.4	240 ± 27	130 ± 37	108 ± 26	2834 ± 645	33 ± 9	41.2	20.3	38.5
Sailing (*n* = 6)	2244 ± 296	9.4 ± 1.2	278 ± 58	101 ± 7	72 ± 5	3670 ± 788	30 ± 2	53.1	18.5	28.4
Gymnastics (*n* = 13)	1566 ± 92	6.6 ± 0.4	199 ± 29	72 ± 8	48 ± 6	1691 ± 136	20 ± 1	53.6	18.9	27.5

Intake values are expressed as mean intake ± SD. Differences between categories are indicated by symbols (one-way ANOVA: *p* < 0.05, with post-hoc Bonferroni correction), each comparison between groups has its own symbol, ★ Endurance vs. Team, ◆ Endurance vs. Strength and ■ Team vs. Strength. No analysis was performed for testing differences between sports within the categories for endurance, team, and strength sports * TE% for CHO includes estimated TE% of dietary fibre.

**Table 4:** Carbohydrate intake (g·kg^−1^) and prevalence (%) of inadequate intake based on sport nutrition recommendation of 5 g·kg^−1^, excluding and including nutritional supplements.

	Men					Women			
	Excluding		Including			Excluding		Including	
	Mean Intake	Low Intakes	Mean Intake	Low Intakes		Mean Intake	Low Intakes	Mean Intake	Low Intakes
**Endurance (*n* = 157)**	**5.1 ± 1.1** ◆	**52.2**	**5.2 ± 1.0** ◆	**48.3**	**Endurance (*n* = 83)**	**4.9 ± 0.9** ★◆	**55.7**	**5.0 ± 0.9** ★◆	**54.4**
Rowing (*n* = 34)	5.7 ± 0.9	38.1	5.8 ± 0.9	36.0	Rowing (*n* = 26)	5.1 ± 0.6	48.5	5.1 ± 0.7	46.9
Swimming (*n* = 11)	5.0 ± 1.4	59.4	5.1 ± 1.4	56.0	Swimming (*n* = 9)	5.2 ± 0.5	45.6	5.4 ± 0.7	43.6
Ice skating (*n* = 15)	5.2 ± 1.0	44.2	5.3 ± 1.0	43.5	Ice skating (*n* = 11)	4.6 ± 1.0	61.4	4.7 ± 1.0	60.9
Road cycling (*n* = 34)	5.4 ± 1.1	42.3	5.6 ± 1.0	38.8	Road cycling (*n* = 14)	4.2 ± 1.1	72.8	4.2 ± 1.2	71.9
Running (mila) (*n* = 8)	6.3 ± 1.4	25.6	6.4 ± 1.4	25.5	Running (mila) (*n* = 11)	5.7 ± 0.9	42.3	5.7 ± 0.9	41.4
Ultra endurance (*n* = 55)	4.2 ± 0.8	71.8	4.5 ± 0.6	64.7	Ultra endurance(*n* = 12)	4.6 ± 0.9	65.9	4.8 ± 1.1	64.0
**Team (*n* = 138)**	**5.0 ± 1.2** ■	**53.4**	**5.1 ± 1.2** ■	**50.5**	**Team (*n* = 104)**	**3.6 ± 0.6** ★	**82.1**	**3.7 ± 0.6** ★	**80.5**
Soccer youth (*n* = 63)	5.7 ± 1.2	38.8	5.7 ± 1.1	38.6	Soccer youth (*n* = 16)	4.3 ± 0.5	68.0	4.4 ± 0.6	64.4
Soccer talent (*n* = 26)	5.0 ± 1.2	49.8	5.2 ± 1.2	47.7	Volleyball (*n* = 18)	3.6 ± 0.4	85.8	3.6 ± 0.4	85.8
Soccer professional (*n* = 30)	4.0 ± 0.5	75.9	4.4 ± 0.6	65.7	Rugby Sevens(*n* = 29)	3.5 ± 0.6	86.1	3.7 ± 0.7	85.0
Water polo (*n* = 12)	4.4 ± 1.3	70.8	4.5 ± 1.4	69.9	Water polo (*n* = 12)	3.5 ± 0.7	83.7	3.5 ± 0.8	80.8
Hockey (*n* = 7)	3.8 ± 0.6	84.1	3.8 ± 0.6	84.1	Hockey (*n* = 11)	4.2 ± 0.5	71.8	4.2 ± 0.5	71.8
					Handball (*n* = 18)	3.1 ± 0.4	91.9	3.2 ± 0.3	91.5
**Strength (*n* = 32)**	**4.1 ± 0.8** ◆■	**74.3**	**4.3 ± 0.8** ◆■	**69.2**	**Strength (*n* = 39)**	**4.1 ± 1.0** ◆	**76.8**	**4.3 ± 1.1** ◆	**73.0**
Track cycling (*n* = 5)	4.2 ± 0.1	76.5	4.5 ± 0.2	66.9	Track cycling/BMX (*n* = 6)	4.0 ± 0.8	80.2	4.1 ± 0.3	76.7
BMX (*n* = 12)	4.7 ± 0.9	57.5	4.9 ± 1.0	52.5	-	-		-	
Sprint/bobsled (*n* = 4)	4.2 ± 0.2	76.5	4.3 ± 0.1	74.1	Sprint/bobsled (*n* = 8)	4.5 ± 0.9	62..8	4.7 ± 0.8	56.7
Cross fit (*n* = 5)	3.2 ± 0.5	94.4	3.4 ± 0.7	87.9	Cross fit (*n* = 6)	3.8 ± 0.5	83.0	3.8 ± 0.5	82.5
Archery (*n* = 6)	3.3 ± 0.8	87.8	3.4 ± 0.9	85.4	-	-		-	
					Sailing (*n* = 6)	3.9 ± 0.5	80.0	4.4 ± 0.9	67.9
					Gymnastics (*n* = 13)	4.1 ± 1.7	79.5	4.2 ± 1.9	79.5

Intake values are expressed as mean ± SD. Prevalence of low intakes (below sports nutrition recommendations of 5.0 g·kg^−1^) are based on the probability approach ([x_i_ − recommendation]/SD_req_) indicating the prevalence of participants *not* meeting recommendations. Differences between categories are indicated by symbols (one-way ANOVAt: *p* < 0.05, with post-hoc Bonferroni correction), each comparison between groups has its own symbol: ★ Endurance vs. Team, ◆ Endurance vs. Strength and ■ Team vs. Strength.

**Table 5:** Protein intake (g·kg^−1^) and prevalence (%) of inadequate intake based on sport nutrition recommendation of 1.2 g·kg^−1^, excluding and including nutritional supplements.

	Men					Women			
	Excluding		Including			Excluding		Including	
	Mean Intake	Low Intakes	Mean Intake	Low Intakes		Mean Intake	Low Intakes	Mean Intake	Low Intakes
**Endurance (*n* = 157)**	**1.5 ± 0.3**	**25.6**	**1.6 ± 0.3**	**23.9**	**Endurance (*n* = 83)**	**1.5 ± 0.2** ★	**23.5**	**1.5 ± 0.2** ★	**21.8**
Rowing (*n* = 34)	1.7 ± 0.2	11.6	1.8 ± 0.3	8.4	Rowing (*n* = 26)	1.5 ± 0.3	20.3	1.6 ± 0.3	17.6
Swimming (*n* = 11)	1.5 ± 0.3	20.9	1.6 ± 0.2	16.2	Swimming (*n* = 9)	1.6 ± 0.2	14.7	1.7 ± 0.2	14.5
Ice skating (*n* = 15)	1.5 ± 0.2	19.4	1.6 ± 0.2	19.4	Ice skating (*n* = 11)	1.4 ± 0.1	27.7	1.4 ± 0.1	27.7
Road cycling (*n* = 34)	1.6 ± 0.3	16.6	1.7 ± 0.3	16.2	Road cycling (*n* = 14)	1.5 ± 0.3	26.4	1.5 ± 0.3	25.8
Running (mila) (*n* = 8)	1.7 ± 0.3	9.3	1.7 ± 0.3	7.9	Running(mila) (*n* = 11)	1.8 ± 0.1	3.1	1.8 ± 0.1	3.1
Ultra endurance (*n* = 55)	1.2 ± 0.1	44.6	1.3 ± 0.1	43.3	Ultra endurance(*n* = 12)	1.3 ± 0.2	48.6	1.3 ± 0.2	43.1
**Team (*n* = 138)**	**1.5 ± 0.2**	**21.5**	**1.6 ± 0.2**	**19.9**	**Team (*n* = 104)**	**1.3 ± 0.2** ★■	**42.4**	**1.3 ± 0.2** ★■	**41.8**
Soccer youth (*n* = 63)	1.5 ± 0.3	25.5	1.5 ± 0.3	23.8	Soccer youth (*n* = 16)	1.3 ± 0.1	32.2	1.3 ± 0.1	32.2
Soccer talent (*n* = 26)	1.6 ± 0.2	16.4	1.6 ± 0.2	15.7	Volleyball (*n* = 18)	1.2 ± 0.1	53.9	1.2 ± 0.1	52.9
Soccer professional (*n* = 30)	1.6 ± 0.2	13.0	1.7 ± 0.2	9.9	Water polo (*n* = 12)	1.1 ± 0.1	69.7	1.1 ± 0.1	69.7
Water polo (*n* = 12)	1.4 ± 0.3	31.5	1.4 ± 0.3	30.5	Rugby Sevens(*n* = 29)	1.4 ± 0.1	31.3	1.4 ± 0.1	30.0
Hockey (*n* = 7)	1.5 ± 0.1	24.4	1.5 ± 0.1	24.4	Hockey (*n* = 11)	1.4 ± 0.1	29.7	1.4 ± 0.1	29.7
					Handball (*n* = 18)	1.3 ± 0.3	47.5	1.3 ± 0.4	47.3
**Strength (*n* = 32)**	**1.5 ± 0.2**	**25.0**	**1.5 ± 0.2**	**24,2**	**Strength (*n* = 39)**	**1.5 ± 0.4** ■	**31.3**	**1.5 ± 0.4** ■	**29.1**
Track cycling (*n* = 5)	1.6 ± 0.2	16.5	1.7 ± 0.2	15.7	Track cycling/BMX(*n* = 6)	1.3 ± 0.3	41.9	1.4 ± 0.3	37.2
BMX (*n* = 12)	1.4 ± 0.3	33.2	1.4 ± 0.3	33.0	-	-		-	
Sprint/bobsled (*n* = 4)	1.5 ± 0.1	12.8	1.6 ± 0.1	10.7	Sprint/bobsled (*n* = 8)	1.4 ± 0.4	42.4	1.4 ± 0.4	36.4
Cross fit (*n* = 5)	1.7 ± 0.2	18.9	1.8 ± 0.2	16.9	Cross fit (*n* = 6)	1.9 ± 0.8	13.9	2.1 ± 0.8	13.0
Archery (*n* = 6)	1.4 ± 0.2	28.9	1.4 ± 0.3	28.9	-	-		-	
					Sailing (*n* = 6)	1.5 ± 0.2	16.2	1.6 ± 0.2	15.5
					Gymnastics (*n* = 13)	1.4 ± 0.3	34.6	1.4 ± 0.3	34.6

Intake values are expressed as mean ± SD. Prevalence of low intakes (below sports nutrition recommendations of 1.2 g·kg^−1^) are based on the probability approach ([x_i_ − recommendation]/SD_req_) indicating the prevalence of participants *not* meeting recommendations. Differences between categories are indicated by symbols (one-way ANOVAt: *p* < 0.05, with post-hoc Bonferroni correction), each comparison between groups has its own symbol: ★ Endurance vs. Team, ◆ Endurance vs. Strength and ■ Team vs. Strength.
